# Therapeutic drug monitoring (TDM) of tyrosine kinase inhibitors (TKI) for optimized outcome in patients with metastatic renal cell carcinoma. The TKI-TDM Trial. Study protocol

**DOI:** 10.2340/1651-226X.2025.43693

**Published:** 2025-06-02

**Authors:** Jakob N. Henriksen, Charlotte U. Andersen, Frede Donskov, Elke Hoffmann-Lücke, Eva Greibe, Niels Fristrup

**Affiliations:** a Department of Clinical Pharmacology, Aarhus University Hospital, Aarhus, Denmark; b Department of Oncology, Aarhus University Hospital, Aarhus, Denmark; c Department of Clinical Medicine, Aarhus University, Aarhus, Denmark; d Department of Forensic Medicine, Aarhus University Hospital, Aarhus, Denmark; e Department of Oncology, University Hospital of Southern Denmark, Esbjerg, Denmark; f Department of Regional Health Science, University of Southern Denmark, Odense, Denmark; g Department of Clinical Biochemistry, Aarhus University Hospital, Aarhus, Denmark

**Keywords:** Metastatic renal cell carcinoma, tyrosine kinase inhibitors, therapeutic drug monitoring, personalized medicine, toxicity management

## Abstract

**Background:**

Metastatic renal cell carcinoma (mRCC) is notably resistant to chemotherapy and radiotherapy. However, tyrosine kinase inhibitors (TKIs) and checkpoint immunotherapy have significantly improved outcomes. Still, about 20% of patients experience disease progression as their best response to TKIs, and 16–63% endure severe toxicities, reducing quality of life. Optimizing dosing is therefore essential. Therapeutic drug monitoring (TDM) is a promising strategy for individualizing treatment. The TKI-TDM trial aims to identify a therapeutic plasma concentration range for six TKIs (axitinib, cabozantinib, pazopanib, sorafenib, sunitinib, tivozanib) in mRCC patients.

**Material and methods:**

This prospective observational study will enroll mRCC patients with at least 6 months of stable disease or regression on TKI therapy. Blood samples will be collected during routine care. Plasma concentrations of TKIs and metabolites will be measured using validated liquid chromatography-tandem mass spectrometry (LC-MS/MS) methods. These levels will be correlated with clinical outcomes including objective response rate, progression-free survival, overall survival, and toxicity. Genetic analysis of *UGT1A1* polymorphisms will explore their influence on pazopanib metabolism and response.

**Interpretation:**

Identifying plasma TKI levels associated with efficacy and reduced toxicity could minimize under- or overdosing, improving outcomes and quality of life. TDM may allow dose adjustments early in therapy, improving therapeutic management and reducing healthcare costs. Findings may also inform treatment of other cancers using TKIs or TKI-immunotherapy combinations. The trial (clinicaltrials.gov NCT04659343) is expected to conclude in 2028, with results in 2029.

## Introduction

Kidney cancer constitutes approximately 5% of all cancers in men and 3% in women [1], and it is notably resistant to chemotherapy and conventional radiotherapy. However, metastatic renal cell carcinoma (mRCC) shows sensitivity to targeted therapy and checkpoint immunotherapy. Targeted therapies for mRCC include tyrosine kinase inhibitors (TKIs) such as sunitinib, pazopanib, cabozantinib, axitinib, sorafenib, and tivozanib. These therapies have significantly improved outcomes for mRCC patients [2, 3]. Nonetheless, around 20% of patients experience disease progression as their best response despite treatment and therefore do not benefit from therapy [4].

The potential benefits of these treatments come with significant side effects. For example, grade 3 toxicity or higher occurs in 16–63% of patients treated with TKIs [4, 5], impacting their quality of life. To manage these side effects, clinicians can reduce doses, temporarily discontinue therapy, or stop treatment permanently. Additionally, underdosing is a clinical issue where patients without side effects continue therapy on an untherapeutic dose level until their next scan, potentially leading to progressive disease due to underdosing. This challenge has led to the consideration of therapeutic drug monitoring (TDM) as a tool to guide individualized therapy.

Clinical experience shows that the optimal dose of TKI varies significantly between patients, in some cases influenced by polymorphisms in the *UGT1A1* gene [6, 7]. Therefore, the prescribed dose alone is not a reliable predictor of treatment outcomes or side effects. Plasma concentration may be a better predictor of these outcomes.

### The potential of TDM in renal cancer management

TDM is an emerging field in oncology, aiming to identify an optimal therapeutic interval on the plasma concentration-response curve. This interval helps clinicians adjust dosing to avoid side effects or increase the dose when levels are too low, ensuring effectiveness. TDM has been successfully applied to immunosuppressant therapy in organ transplant patients, anticoagulant therapy and patients treated with lithium [8].

TKIs meet traditional criteria for TDM use, including long-term and continuous therapy, available bioanalytical methods, significant inter-individual but limited intra-individual pharmacokinetic variability, and consistent associations between concentration and response [9]. However, research in this area is limited [10].

Current treatment guidelines recommend immunotherapy combinations immuno-oncology (IO) (IO-IO) or TKI-IO combinations as first-line treatments, in addition TKIs are used as monotherapy in the International Metastatic mRCC Database Consortium (IMDC) favorable risk group in some countries, in frail patients, and in subsequent lines of therapy [11]. Studying TKI-only treated patients will likely provide robust scientific data. Understanding the role of plasma concentrations of TKIs will in turn provide knowledge on combination regimens and thereby individualize and optimize these treatments even further.

Given the high cost of these treatments, more precise dosing could represent an economic benefit for public healthcare systems. The findings in the TKI-TDM trial may also be relevant for other cancer types as the use of TKIs expands.

### Rationale

We assume that patients treated with TKIs for more than 6 months with computed tomography (CT)-verified stable disease or disease regression have optimal plasma trough concentrations, and that objective response rate, progression free survival (PFS), overall survival (OS), and toxicity in these patients are similar to patients treated in pivotal phase III study.

### Hypothesis

The TKI-TDM trial hypothesizes that it is possible to elucidate a therapeutic interval for the TKIs examined in the study

### Aim

To compare the trough plasma concentrations of TKIs in patients with mRCC having stable disease with those experiencing toxicity, treatment failure, and stable disease or regression, respectively to identify the optimal plasma concentration level.

## Material and methods

### Design

The study is a prospective non-intervention observational trial conducted at Aarhus University Hospital from March 1, 2022 to August 31, 2028. All consecutive patients with mRCC treated with monotherapy of a TKI in the study period will be asked to participate in the study.

### Inclusion criteria

- Patients with mRCC receiving axitinib, cabozantinib, pazopanib, sorafenib, sunitinib, or tivozanib in monotherapy after reaching 6 months of stable disease or disease regression evaluated by the assessor.
- Patients aged 18 years or older.
- Patients must be able to understand and sign the written informed consent form.

### Exclusion criteria

- Patients who do not provide written informed consent.
- Patients unlikely to comply with the protocol (e.g. uncooperative attitude), unable to return for subsequent visits, and/or otherwise considered to be unlikely to complete the study by the investigator.
- Patients on combination therapy.

### Protocol

**Figure 1:**
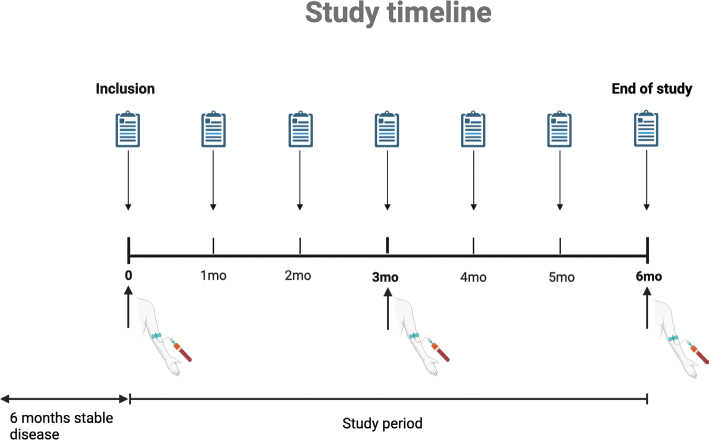
Timeline of the study. Paper sheets represent the FKSI-19 questionnaire and syringes represent blood samples. Each intervention will be performed at the corresponding time point in the study period, that is, questionnaires each month and blood samples every third month.

Participants will give blood samples during their regular visits to the clinic for 6 months after inclusion. Before each scheduled clinical visit, patients will have routine blood tests and an additional 40 ml of blood will be collected for analysis (Figure 1). Patients will take their medication the day before the visit, but not on the day of the blood sampling.

### Concentration measurements

Plasma concentrations of TKIs and metabolites will be determined using a validated liquid chromatography-tandem mass spectrometry (LC-MS/MS) method at the accredited laboratory at the Department of Clinical Biochemistry, Aarhus University Hospital. The method follows ISO15189 principles with a precision and bias lower than 15%. Protein precipitation will be performed with methanol and stable labeled isotope internal standards will be used to correct for matrix effects during analysis. Mass spectrometry has previously proven effective in measuring the plasma concentrations of axitinib [12], sorafenib [13], sunitinib [14], pazopanib [15], and its metabolites [16]. Of the six TKIs, only sorafenib and sunitinib have active metabolites (sorafenib N-oxide and desethylsunitinib, respectively) [17–22]. The parent drug will therefore be measured for all TKIs with the addition of sorafenib N-oxide and desethylsunitinib when relevant.

### Genetic analysis

The *UGT1A1* gene analysis will use in-house expertise at the Department of Clinical Biochemistry, Aarhus University Hospital. The human *UGT1A1* gene (NG_033238.1) will serve as a template for the design of the primers, and the nucleotide sequence has already been obtained from the National Centre for Biotechnology Information platform (available at: https://www.ncbi.nlm.nih.gov/). Standard polymerase chain reaction (PCR) amplification will be performed using forward primer 50-FAM GCTCCACCTTCTTTATCTCTGAAAGTGAACT- 30 and reverse primer 50-CTAACAAAAGACTCTTTCACATCCTCCCTTTGGA- 30. After PCR amplification, fluorescein amidite labeling allows for the detection of 448- to 450-bp fragments, which corresponded to 6 and 7 TA repeats, respectively. This analysis will be performed using the Applied Biosystems 3500 Genetic Analyzer mounted with a 50-cm capillary (Applied Biosystems, ThermoFisher Scientific, Waltham, MA) using POP7 polymer and the GeneScan 500 ROX dye size standard (35–500 bp; Applied Biosystems) according to the manufacturer’s instructions.

Analysis of TKIs and metabolites will be done at the Department of Clinical Biochemistry, Aarhus University Hospital.

### Status

Inclusion began in March 2022, was paused for almost 2 years due to COVID-19, and is expected to end in August 2028. The therapeutic drug intervals are expected to be ready for publication in 2029. The study is registered in the Clinical Trial Database: NCT04659343.

### Endpoints and analysis

The TKI-TDM trial will evaluate the accuracy and clinical usability of TDM using clinical endpoints: measured drug concentrations will be correlated to objective best response, PFS, OS, and toxicity. Response will be categorized according to RECIST 1.1 criteria [23], and survival analysis will use the Kaplan-Meier method and log-rank test.

To assess side effects, the Functional Assessment of Cancer Therapy-Kidney Symptom Index (FKSI)-19 score [24] will be used, provided to patients via an electronic questionnaire via REDCap. FKSI-19 has been validated in patients with mRCC and used in randomized phase 3 trials to assess quality of life.

### Ethical considerations, risks and side effects

Most blood samples are part of routine practice, and additional samples for the study pose minimal risk, primarily local pain, bleeding, or infection from blood sampling. Results from the analyses will not influence the individual patient’s clinical management. Thus, dosing or treatment choice of study participants will be unaffected by either study analyses or results. The study will follow ethical guidelines, ensuring compliance with the Helsinki Declaration and Danish laws. The study is approved by the Central Denmark Region Committees on Health Research Ethics (j. no. 1-10-72-277-19).

### Data protection

The study will comply with the Data Protection Act and the General Data Protection Regulation (GDPR). The study is registered at the Central Denmark Region, Aarhus University Hospital and clinicaltrials.gov (NCT04659343). It will adhere to the Helsinki Declaration and Danish laws on clinical trials and data protection.

### Publication of results

Results, whether positive, negative, or inconclusive, will be published in peer-reviewed journals following the Vancouver criteria.

## Discussion

This study will contribute to the limited knowledge regarding plasma concentration ranges of TKIs used in mRCC treatment [25]. As an observational study, it permits patients to continue their current best-practice therapies, with the only intervention being the collection of an additional blood sample during routine testing. These features are expected to facilitate a higher inclusion rate.

Although TKI-IO combination therapy is also recommended as first-line therapy [26], excluding individuals on combination therapy excludes confounding effects from additional drugs. Findings from this study on TKIs in monotherapy are likely to inform future research on plasma concentration ranges in combination therapy as well.

One limitation of this study is the timing and number of blood samples collected. To estimate the trough plasma concentration (Cmin), patients are asked to withhold their morning dose before sampling. While alternative pharmacokinetic measures, such as area under the curve (AUC) or maximum plasma concentration (Cmax) are reported in other studies, Cmin was selected as it is the most commonly reported parameter in TDM trials [25] and offers the greatest clinical applicability for dose titration if a therapeutic interval is established.

Another limitation is the potential underrepresentation of patients experiencing toxicity, as this study includes only patients with stable disease. This approach ensures that their plasma concentrations are most likely within the therapeutic range, as unstable patients would typically exhibit intolerable side effects, disease progression, or both. However, during the 6-month study period, some participants are anticipated to develop side effects or experience disease progression, enabling comparisons of their plasma concentrations with those of patients maintaining stable disease and tolerable side effects.

We chose to abstain from dense sampling, which involves intensive blood collection over a short interval (e.g. within 24 h post-dose), to enhance comparability with existing research.

Although there is no immediate benefit for participants, the study aims to optimize future treatment strategies, potentially applicable across cancer types, thereby improving outcomes and reducing healthcare costs. From a societal perspective, early identification of optimal dosing and treatment strategies could significantly decrease medical expenditures and improve patients’ quality of life.

### Perspectives

Determining a therapeutic interval for TKIs could optimize treatment for mRCC patients by:

1. Titrating up doses for patients with insufficient levels to reduce cancer progression risk.
2. Titrating down doses for overdosed patients to limit toxicity and costs.
3. Monitoring patients on intermittent therapy interacting with TKI treatment.

These results may extend to other cancer types using TKIs.

## Conclusion

In this prospective study, the TKI-TDM trial, patients with mRCC treated with TKIs in monotherapy, and after reaching a phase of stable disease or disease regression will provide extra blood samples for a 6 months period to elucidate the role of plasma concentration in treatment efficacy and side effects. This may contribute to the identification of a therapeutic interval, aiding the treatment with TKIs, potentially increasing treatment efficacy, and reducing side effects. Furthermore, this has the potential to optimize the treatment of TKI-IO combinational therapy as well.

## Data Availability

The datasets used and/or analyzed during the current study will be available from the corresponding author on reasonable request.
